# ELF5‐Mediated Enhancer–Promoter Interaction Regulates *LALBA* Expression in Ovine Mammary Gland

**DOI:** 10.1002/advs.78138

**Published:** 2026-10-04

**Authors:** Xinmiao Wu, Jiqing Wang, Zhiyun Hao, Mingna Li, Huimin Zhen, Yuan Liu, Guoyong Zhao, Chunyan Ren, Xuelan Zhou, Haixiong Hu, Mingjie Li, Nan Shi

**Affiliations:** ^1^ Gansu Engineering Lab of Genetic Improvement in Ruminants, College of Animal Science and Technology Gansu Agricultural University Lanzhou China

**Keywords:** chromatin accessibility, epigenetic regulation, lactation, *LALBA* super‐enhancer

## Abstract

Epigenetic mechanisms underlying lactation traits in sheep remain poorly understood. Here, we systematically characterized the transcriptomic dynamics, chromatin accessibility landscape, and histone modification profiles of mammary gland tissues from Yuansheng Aite dairy sheep with divergent lactation performance. The lactalbumin alpha (*LALBA*) locus harbors a super‐enhancer, and expression of *LALBA* was upregulated in sheep with low lactation performance. Enhancer E4 functions as the core active element within the *LALBA* super‐enhancer, and its repression reduced *LALBA* expression. Chromosome conformation capture‐polymerase chain reaction (3C‐PCR) confirmed a direct chromatin interaction between E4 and the *LALBA* promoter. The E74‐like ETS transcription factor 5 (ELF5) binds to both E4 and the *LALBA* promoter, consistent with the observation that E4 and the *LALBA* promoter region exhibit greater chromatin accessibility in the low lactation ewes. E4 promotes *LALBA* transcription via ELF5‐mediated chromatin looping, thereby inhibiting cell cycle progression and the proliferation of ovine mammary epithelial cells (OMECs). This study suggests a new target and provides a theoretical basis for the genetic improvement of lactation performance in sheep.

## Graphical abstract

In the study, we systematically investigate the epigenetic mechanisms underlying lactation traits in sheep by integrating transcriptomic, chromatin accessibility, histone modification, and functional analyses. We identify a super‐enhancer at the *LALBA* locus and demonstrate that enhancer E4 functions as its core regulatory element. Furthermore, ELF5 facilitates enhancer‐promoter chromatin looping to activate *LALBA* transcription.

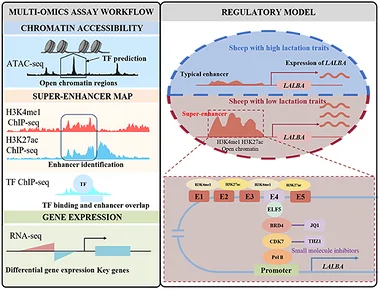

## 1 Introduction

Dairy products provide valuable nutrition and constitute an important part of the human diet [1, 2]. Compared with the milk of other ruminants, sheep milk contains higher concentrations of omega‐3 fatty acids [3] and has a higher dry matter content [4]. Sheep milk also contains higher levels of α‐ketoglutarate and myo‐inositol than goat milk, and both compounds may confer health benefits [5]. In contrast to cow milk and goat milk, sheep milk is characterized by typically has a lower production yield and higher market price [6, 7, 8, 9]. Accordingly, improving milk‐production performance would enhance the economic benefits of the sheep industry.

Gene expression in the mammary gland regulates cell proliferation and apoptosis, thereby influencing lactation performance [10, 11]. The primary regulators of gene expression are enhancers [12, 13]. They regulate transcription by establishing physical interactions with the promoters of target genes to form chromatin loops and by recruiting specific transcription factors (TFs) and cofactors to gene promoters, thereby leading to transcriptional activation [14, 15, 16].

Chromatin Immunoprecipitation sequencing (ChIP‐seq) is the preferred method for genome‐wide mapping of TF binding sites and epigenetic marks [17, 18, 19, 20]. Monomethylation of histone 3 lysine 4 (H3K4me1) is widely regarded as a canonical marker for identifying enhancers across the genome [21, 22, 23]. The coexistence of H3K4me1 and acetylation of lysine 27 on histone 3 (H3K27ac) generally indicates an active enhancer [24, 25, 26]. Deciphering the interplay between chromatin accessibility and binding of TF or these marks is fundamental to understanding transcriptional regulation, the control of cellular states, and the establishment of novel phenotypes [27, 28]. Such analyses may therefore be suitable for gaining a better understanding of the genetic mechanisms underlying differences in lactation performance.

Existing studies reflect a scarcity of validated enhancers known to modulate lactation phenotypes [29]. Lee et al. [30] characterized three mammary gland‐specific enhancers located upstream of the β‐casein (CSN2), and showed that expression was reduced by approximately 50% following deletion of all three enhancers. Furthermore, knockout of the proximal enhancer of the kappa‐casein (*CSN3*) in mouse mammary gland tissue abrogated the binding site of signal transducer and activator of transcription 5 (STAT5) within the enhancer, resulting in a 98% reduction in *CSN3* expression. The high‐level, tissue‐specific expression of the whey acidic protein (*WAP*) in mice depends on highly selective lineage‐specific synergy between its super‐enhancer and its native promoter [31]. Despite these advances, the comprehensive landscape and functional characteristics of mammary gland‐associated enhancers in sheep have not been systematically explored, and the dynamics of enhancer‐promoter interactions in modulating lactation performance remain unclear.

In the current work, we first performed a genome‐wide analysis of chromatin accessibility in ovine mammary gland tissues, and identified potential TFs that drive the process of mammary gland development. Furthermore, we investigated the binding sites of E74‐like ETS transcription factor 5 (ELF5), H3K4me1 and H3K27ac in the ovine mammary gland, and the super‐enhancer map was constructed accordingly. Subsequently, we identified an enhancer termed E4, that recruits ELF5 to physically interact with the promoter of the lactalbumin alpha (*LALBA*) by forming an enhancer‐promoter chromatin loop to promote *LALBA* transcription. This process ultimately influences the cellular characteristics of ovine mammary epithelial cells (OMECs). Our findings therefore describe an epigenetic mechanism that regulates lactation performance in sheep and provides new insights into the transcriptional regulation of ovine lactation.

## 2 Results

### 2.1 Microstructure Characteristics of Ovine Mammary Gland

The microstructure characteristics of the mammary gland are correlated with the milk production performance of ewes. Therefore, an investigation was conducted to ascertain whether there were differences in the mammary gland microstructure between high lactation performance ewes and low lactation performance ewes. Abundant mammary gland acini can be observed within the lobules of the mammary gland, and these acini were round or nearly round in shape, which were constituted by single‐layered mammary gland epithelial cells. Notably, there was a greater amount of pinkish secretion‐milk in the acinar cavities of high lactation performance ewes (Figure 1A). In consideration of the size of acinus determine the milk yield and milk composition of ewes, the area, diameter and circumference of acinus cavity were counted. It was observed that the area, diameter and circumference of acinus cavity of ewes with high lactation performance were greater than those of ewes with low lactation performance (*p* < 0.05) (Figure 1B).

**FIGURE 1:**
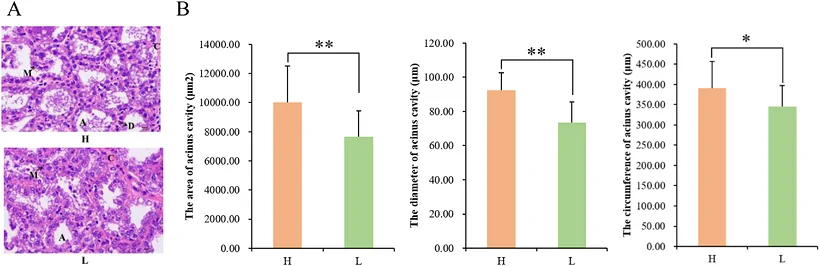
Hematoxylin‐eosin (H&E) staining (A) and statistical analysis (B) of mammary gland parenchyma from Yuansheng Aite dairy sheep with high (H) and low (L) lactation performance. Scale bar = 10 µm. C: connective tissue; D: ducts; A: alveolar lumen; M: mammary gland epithelial cells. Data are represented as mean ± SD (*n* = 9). Statistical significance was determined by two‐tailed unpaired *t*‐test. *p < 0.05 and **p < 0.01.

### 2.2 Genome‐Wide Chromatin Accessibility Landscape of Ovine Mammary Gland

The quality of each ATAC‐seq library was tested, and it was detected that the distribution of ATAC‐seq library fragments was consistent. Briefly, the length of DNA fragments mainly concentrated in the range of 100–800 bp. ATAC‐seq reads were mainly enriched in the vicinity of transcriptional start site (TSS) (Figure S1A), indicating successful enrichment of accessible chromatin regions (ACRs) and high‐quality sequencing libraries. We identified 30673 and 19942 ACRs in mammary gland of ewes with high lactation performance and low lactation performance, respectively (File S1). The peaks were mainly concentrated in promoter, intron and intergenic regions of the whole genome, and these peaks had the highest abundance were observed in promoter regions (Figure S1B), which reached 36.34% and 45.94% of all peaks in ewes with high lactation performance and low lactation performance, respectively (Figure S1C). Most of the peaks were located in 0–1 and 10–100 kb regions relative to TSS of their nearest genes (Figure S1D), which was consistent with the distribution of peaks in functional areas.

A total of 33546 non‐redundant consensus ACRs were obtained by merging ACRs identified in the high and low lactation performance groups (File S2). Based on differential accessibility analysis, there were 215 peaks had significantly increased ACRs in the mammary gland of high lactation performance ewes, while there were 280 peaks had significantly decreased ACRs in the tissue (Figure 2A). The differential ACRs were mainly distributed in intergenic and intron regions of genome (Figure 2B), indicating that the gene transcription level of ovine mammary gland may be more regulated by the distal cis‐regulatory element. Differential peaks mainly distributed in the range of 10–100 kb (49.69%) and >100 kb (30.71%) of TSS of their nearest genes (Figure 2C), and this also proved that changes of gene transcription may be more regulated by distant regulatory regions. The genes of differential ACRs were enriched in various biological functions by Gene Ontology (GO) analysis, including the terms of negative regulation of T cell proliferation and multicellular organism development that have been proved to regulate mammary gland development (Figure 2D). The Kyoto Encyclopedia of Genes and Genomes (KEGG) results showed that the differential ACRs related genes were significantly related to insulin resistance, aldosterone synthesis and secretion and apelin signaling pathway, these pathways were related to milk composition (Figure 2E).

**FIGURE 2:**
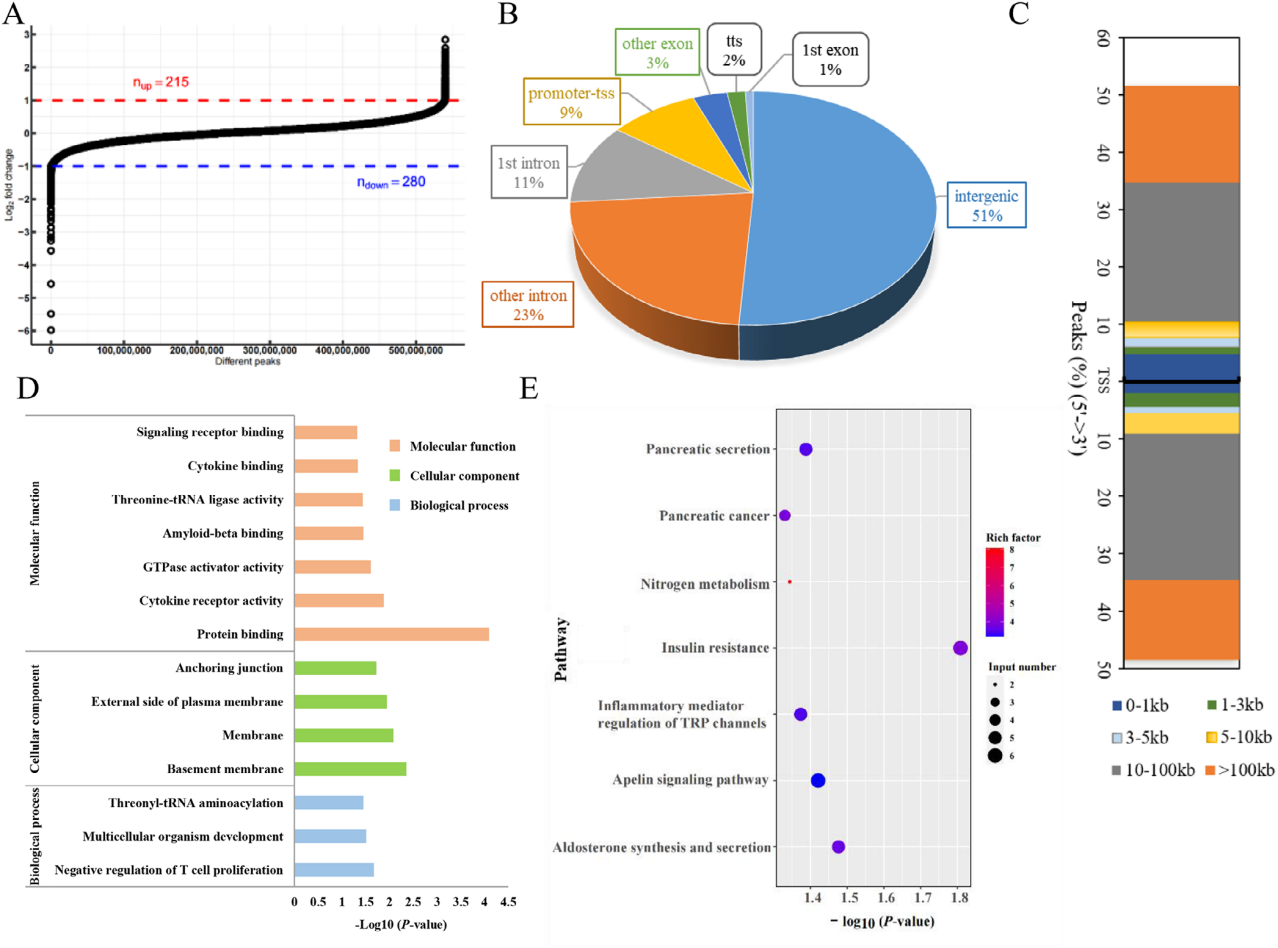
Differential accessible chromatin regions (ACRs) analysis of Yuansheng Aite dairy sheep with high lactation performance and low lactation performance. (A) The number of differential peaks in ovine mammary gland. (B) The distribution of the genomic location of differential peaks. (C) Proportion of peaks distributed upstream and downstream of the transcription start site (TSS) for genes of differential ACRs. (D) GO analysis of the upregulated and downregulated ACRs related genes. (E) The KEGG pathways of the upregulated and downregulated ACRs related genes.

### 2.3 Chromatin Accessibility Driven Gene Expression in Ovine Mammary Gland

A total of 765 differentially expressed genes (DEGs) were identified in the ovine mammary gland between high lactation performance ewes and low lactation performance ewes using RNA‐seq. Of these, 296 genes were up‐regulated, while 469 genes were down‐regulated in mammary gland of high lactation performance ewes compared to low lactation performance ewes (File S3). The DEGs were significantly enriched in 26 biological process terms, 11 cellular components terms and 12 molecular function terms. It was discovered that several significant GO terms were directly related to mammary gland development and lactation. For example, up‐regulated genes were related to activation of NF‐kappa B‐inducing kinase activity, lipid metabolic process and lipid transport (Figure 3A). Down‐regulated genes were participated in lactose biosynthetic process and fatty acid biosynthetic process (Figure 3B). Up‐regulated genes were mainly enriched in lysosome, regulation of lipolysis in adipocytes, and phospholipase D signaling pathway (Figure 3C). While down‐regulated genes were associated with glutathione metabolism, sulfur metabolism, and galactose metabolism (Figure 3D). A total of 12 DEGs were randomly selected to validate the reliability of RNA‐seq data. Reverse transcription‐quantitative polymerase chain reaction (RT‐qPCR) validation showed expression tendency consistent with those obtained from the RNA‐seq analysis (Figure 3E).

**FIGURE 3:**
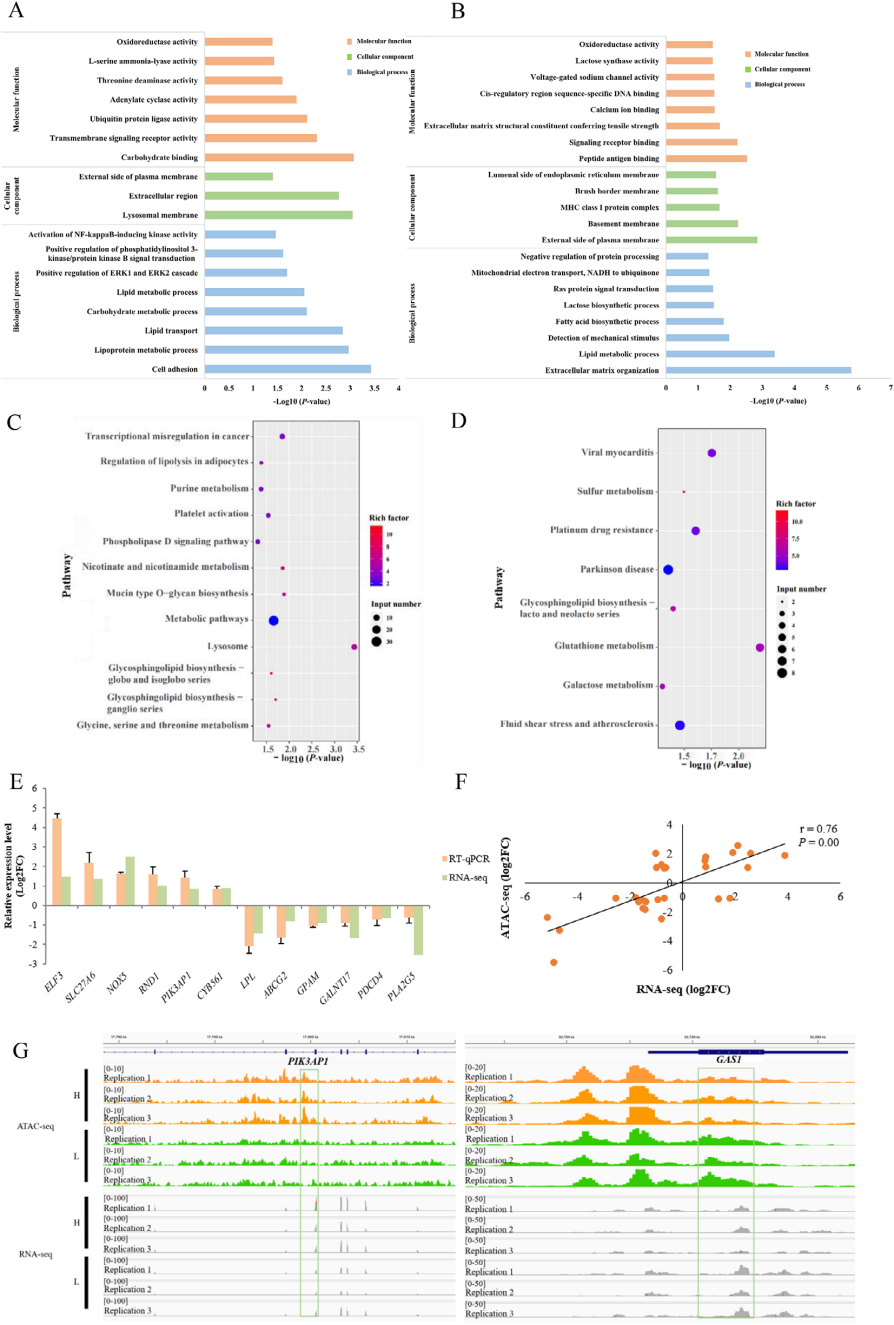
The relationship between chromatin accessibility and gene expression obtained from RNA‐seq. GO analysis of the up‐regulated (A) and down‐regulated genes (B) identified in ovine mammary gland between the two groups. KEGG analysis of the up‐regulated (C) and down‐regulated genes (D) identified in ovine mammary gland. Up‐regulated and down‐regulated means higher and lower expression in the high (H) lactation performance ewes compared to low (L) lactation group, respectively. (E) RT‐qPCR validation of DEGs in mammary gland using RNA‐seq. (F) Correlation analysis results between chromatin openness and gene expression. Each dot represents accessible chromatin regions (ACRs)‐gene pair, with the x‐axis showing the log_2_ Fold Change in gene expression and the y‐axis showing the corresponding log_2_ Fold Change in chromatin accessibility. (G) Visualization of DEGs driven by ACRs in mammary gland of H and L groups. The peaks are ACRs shown to provide genomic context, whereas the boxed regions were selected as representative differentially ACRs identified by ATAC‐seq, together with their corresponding regions in the RNA‐seq tracks.

We examined the relationship between chromatin accessibility and gene expression in ovine mammary gland by integrating analysis results of RNA‐seq and ATAC‐seq (File S4). The results showed that there was a strong positive correlation between differential ATAC‐seq peaks and gene expression levels obtained from RNA‐seq (Figure 3F). A total of 29 genes were screened by overlapping DEGs with genes of differential ACRs. Notably, phosphoinositide‐3‐kinase adaptor protein 1 (*PIK3AP1*) was up‐regulated and contained increased ACRs in mammary gland of high lactation performance ewes, and the gene was involved in the PI3K‐Akt signaling pathway. Growth arrest specific 1 (*GAS1*) was down‐regulated and contained decreased ACRs in mammary gland of high lactation performance ewes, which induced cell apoptosis in mammary gland (Figure 3G). These findings demonstrated that chromatin accessibility regulated the gene expression in ovine mammary gland.

### 2.4 ELF5 as the Most Highly Expressed ETS Member and its Binding Sites in Ovine Mammary Gland Were Identified

To identify the potential TFs underlying differences in chromatin accessibility between the two groups, TF‐binding motif enrichment analysis was performed on differential ACRs in ovine mammary gland tissues. Motif analysis based on the differential peaks identified a total of 114 motifs, of which 71 were known motifs and 43 were novel motifs. The most of these TFs belong to E26 transformation‐specific (ETS) family. The top 10 known motifs are shown in Figure 4A, and a full list of motifs can be found in File S5. To evaluate the significance of ETS family across mammary gland, the expression levels of all ETS family members in RNA‐seq data obtained from the mammary gland were analyzed. We found ELF5 to be the most highly expressed ETS family member in mammary gland tissue (Figure 4B). The epithelium‐specific ETS TFs (ESEs) are a subfamily of ETS TFs, including ELF3, ELF5, EHF and SPDEF. The relative expression level of *ELF3*, *ELF5* and *EHF* were further detected by RT‐qPCR, while the expression of *SPDEF* in mammary gland was not be detected because it was not expressed in our RNA‐seq data (Figure 4C). We identified a greater number of footprints of three ESEs family members *ELF3*, *ELF5* and *EHF* in the mammary gland, and the three members had very similar DNA‐binding motifs in ACRs. The signal intensity appears higher in the high lactation group, suggesting greater local chromatin accessibility around these motifs in high lactation ewes (Figure 4D).

**FIGURE 4:**
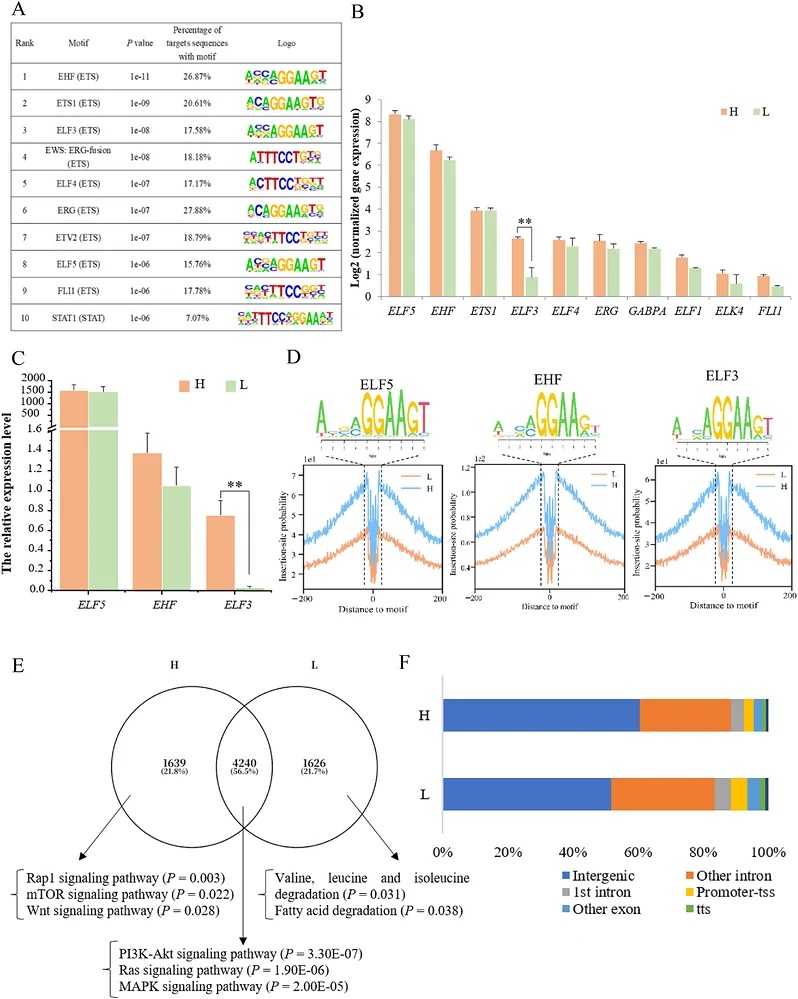
Identification of transcription factors (TFs) and analysis of ELF5 genomic binding in mammary gland of high (H) and low (L) lactation performance ewes. (A) Top 10 known motifs of the differential peaks between the two groups. (B) The top 10 TFs in terms of expression levels in the E26 transformation‐specific (ETS) family (*n* = 3). (C) The expression of three epithelium‐specific ETS (ESEs) family members detected by RT‐qPCR (*n* = 3). (D) Accumulated footprint plots for TFs of three ESEs family members in ovine mammary gland. (E) Functional annotation analysis results of the target genes closest to ELF5 peaks was shown by Venn diagrams. (F) Distribution of ELF5 peaks in different genomic functional regions. Data are represented as mean ± SD. Statistical significance was determined by two‐tailed unpaired *t*‐test. **p < 0.01.

To elucidate the mechanism by ELF5 regulates lactation production performance in sheep, we performed ChIP‐seq to identify ELF5 binding sites across the genome using ELF5 antibody on the same tissues as those used for the ATAC‐seq analysis. ELF5 peaks were more abundant in ovine mammary gland of high lactation performance ewes compared to low lactation performance ewes. A total of 61118 ELF5 peaks were identified in mammary gland of high lactation performance ewes, while 33750 ELF5 peaks were identified in low lactation performance ewes, these regions may represent putative ELF5‐binding regions (File S6). The genes associated to the nearest ELF5 peak were intersected with the genes expressed in the mammary gland obtained by RNA‐seq to get the target genes of ELF5. There were 1639 and 1626 specific target genes of ELF5 for high lactation performance ewes and low lactation performance ewes, respectively. A total of 4240 target genes were shared by the two groups. Functional annotation analyses showed that the target genes of ELF5 identified in ovine mammary gland were related to mammary gland development and lactation performance (Figure 4E). Analysis of the distribution of ELF5‐binding sites relative to annotated TSS revealed that 57.43% and 29.64% of ELF5‐bound regions were intergenic and intron sites, respectively (Figure 4F). Binding at intergenic and intronic regions could represent in some cases binding to regulatory elements such as enhancers.

### 2.5 Super‐Enhancers are Enriched in the Mammary Gland of Sheep

As demonstrated above, a substantial proportion of differential ACRs and ELF5‐binding sites were located in intronic and intergenic regions (Figure 2B and Figure 4F), which harbor numerous enhancer regulatory elements. Collectively, these observations highlight that enhancers represent the most critical regulatory elements involved in the regulation of ovine lactation traits. To further clarify the underlying molecular mechanisms regulating ovine lactation traits, we conducted systematic identification and comprehensive analysis of enhancers. We identified an average of 22253 and 20133 potential enhancers in mammary gland tissues of sheep with high lactation traits and low lactation traits, respectively (File S7). A total of 10239 and 9485 typical enhancers, as well as 682 and 608 super‐enhancers were found in high lactation traits ewes and low lactation traits ewes, respectively. The average length of the super‐enhancers was 7.5‐fold greater than the typical enhancers (Figure 5A).

FIGURE 5Characterization of super‐enhancers of the mammary gland of sheep with high (H) and low (L) lactation performance. (A) The H3K27ac ChIP‐seq peaks in mammary gland were used for analyzing super‐enhancers and typical enhancers using Ranking Of Super Enhancer (ROSE). The inflection point in the rank‐order plot was used to distinguish super‐enhancers from typical enhancers, and regions above the inflection point of the rank‐order curve were defined as super‐enhancers. (B) Genomic distributions of the typical enhancers and super‐enhancers. (C) Representative integrative genomics viewer (IGV) tracks of the typical enhancers and super‐enhancers related genes identified separately in the H and L groups. The small bars below the tracks indicated typical enhancers and the large bars indicated super‐enhancers. (D) Aggregate plots of H3K27ac and H3K4me1 modifications from typical enhancers (top) and super‐enhancers (bottom) identified separately in the H and L groups.

**Figure:**
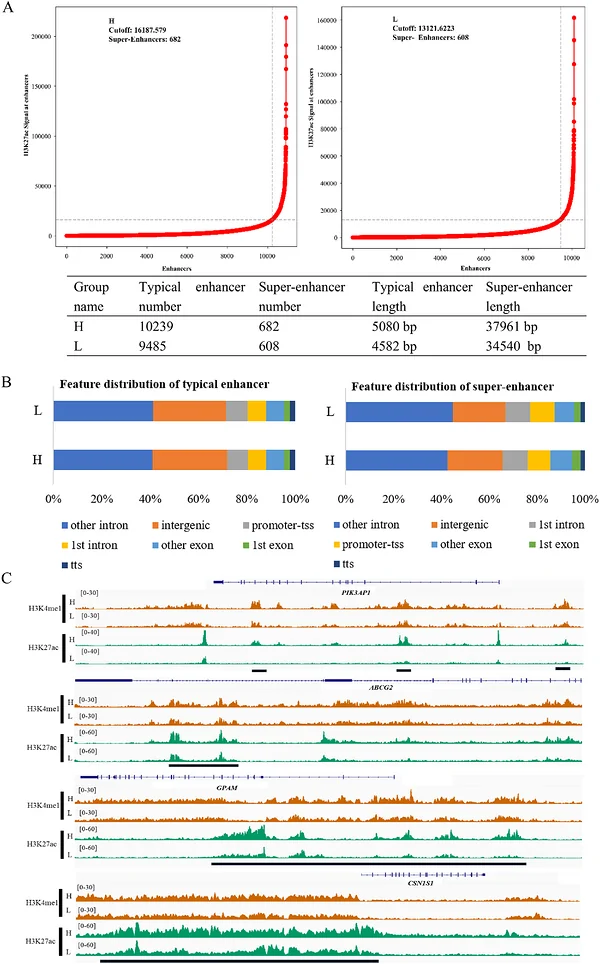

**Figure:**
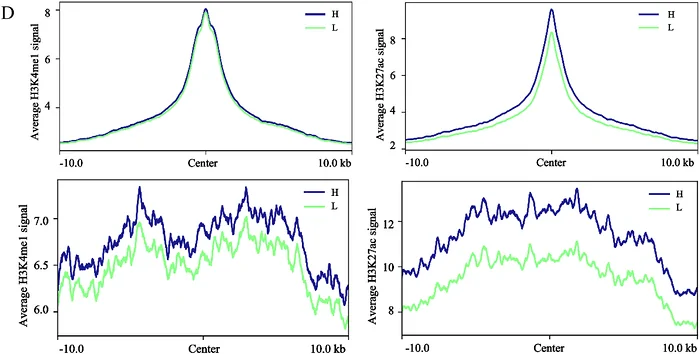

The genome distribution of typical enhancer and super‐enhancer was similar, and they were mostly located in introns and intergenic regions (Figure 5B). Representative tracks from one of the three biological replicates for the typical enhancers and super‐enhancers are shown in Figure 5C. To assess the functional relevance of super‐enhancers in mammary gland tissue, we compared the expression of genes associated with super‐enhancers to those genes associated with typical enhancers based on RNA‐seq data. The expression level of super‐enhancer‐associated genes in mammary gland tissue showed higher average expression than genes associated with typical enhancers in both groups (File S8). Super‐enhancers had higher H3K27ac signals than typical enhancers, and the span of super‐enhancers was larger (Figure 5D) (File S9).

### 2.6 A Novel Super‐Enhancer of *LALBA* was Identified in Mammary Gland

As super‐enhancers specifically drive high transcriptional of genes, we hypothesized that combining super‐enhancers profiles with gene expression data derived from same samples would allow us to pinpoint novel genes that are critically regulated in lactation performance. To this end, the group‐defined super‐enhancers were identified, there were 29 super‐enhancers were defined in high lactation ewes but not observed in low group, whereas 26 super‐enhancers were defined in low lactation ewes but classified as typical enhancers in high lactation ewes (Figure S2A). Importantly, *LALBA* super‐enhancer only defined in low lactation performance ewes, but it was a typical enhancer in high lactation performance ewes. Moreover, *LALBA* was significantly down‐regulated at both the transcript and protein levels in high lactation performance ewes (Figure 6A). To investigate whether the high expression of *LALBA* was due to activation of super‐enhancers, we treated OMECs with small molecule JQ‐1 and THZ1, which has been demonstrated to specifically inhibit super‐enhancers associated genes expression. JQ‐1 and THZ1 treatment dramatically decreased the expression of *LALBA* (Figure S2B). In summary, we discovered *LALBA* expression was associated with super‐enhancer.

**FIGURE 6:**
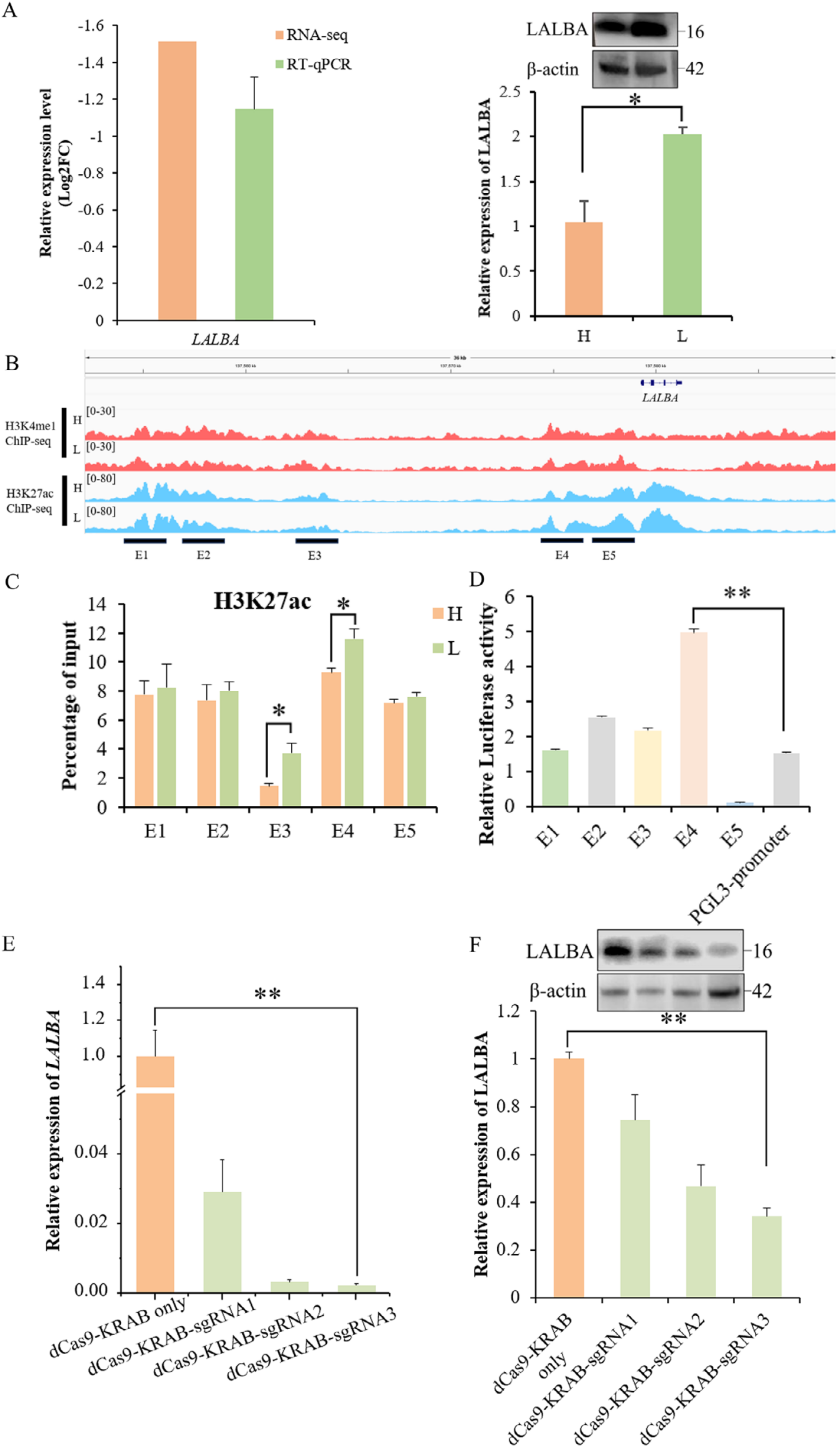
Characterization and activity verification of *LALBA* super‐enhancer. (A) Expression of *LALBA* in mammary gland of high (H) and low (L) lactation ewes at mRNA and protein level. Representative images from three independent biological replicates are shown (Original uncropped blots can be found in Supplementary figure: “Original blots for (A)”). (B) Visualization of active histone marks at the *LALBA* super‐enhancer. (C) H3K27ac enrichment at enhancers of *LALBA* assessed by ChIP‐qPCR. (D) The activity of enhancers validated via luciferase assay. The *LALBA* mRNA (E) and protein expression (F) was determined when E4 activity was repressed using dCas9‐KRAB‐sgRNA. The “dCas9‐KRAB only” does not include an sgRNA. Original uncropped blots can be found in Supplementary figure: “Original blots for (F)”. Data are represented as mean ± SD (*n* = 3). For statistical analysis, two‐tailed unpaired Student's *t*‐tests were performed in A and C; one‐way ANOVA plus Tukey's post hoc tests were performed in (D–F). *p < 0.05, **p < 0.01.

*LALBA* super‐enhancer consists of five typical enhancers named E1–E5, which were based on H3K27ac and H3K4me1 enrichment in a 23 kb region along the *LALBA* locus either side of the TSS (Figure 6B). ChIP‐qPCR confirmed that the modification level of H3K27ac of E3 and E4 in high lactation performance ewes was lower than that in low lactation group, and E4 exhibited the highest level of H3K27ac enrichment (Figure 6C). We cloned five enhancers with prominent ATAC‐seq peaks into a dual‐luciferase reporter vector, and revealed that E4 had the highest activity (Figure 6D). We utilized a dCas9‐KRAB/sgRNA system which could be used to repress the enhancer activity of E4. Transfection of three different sgRNAs were significantly repressed the expression of *LALBA* (Figure 6E; *p* < 0.01). The inhibitory effect of *LALBA* expression was alleviated after overexpressing *LALBA* (Figure S2C). These findings definitively demonstrate E4 is a core enhancer within the *LALBA* super‐enhancer and directly regulates *LALBA* transcription.

### 2.7 E4 Induced *LALBA* Transcription by Forming Chromatin Loops and Altered the Characteristics of OMECs

To explore whether key region of super‐enhancer‐E4 could directly interact with *LALBA* promoter, we performed 3C‐PCR to determine enhancer‐promoter loop formation. 3C analyses demonstrated a direct three‐dimensional interaction at the chromosome level between E4 and *LALBA* promoter, suggesting the regulatory role of E4 might be exercised through direct engagement with *LALBA* promoter (Figure 7A). To understand how the spatial proximity of E4 to *LALBA* promoter boosts the transcription of *LALBA*, the E4 was cloned into luciferase reporter vectors containing the *LALBA* promoter, and we found that E4 can improve the activity of the promoter to the greatest extent compared to other enhancers (Figure 7B, S3; *p* < 0.01).

**FIGURE 7:**
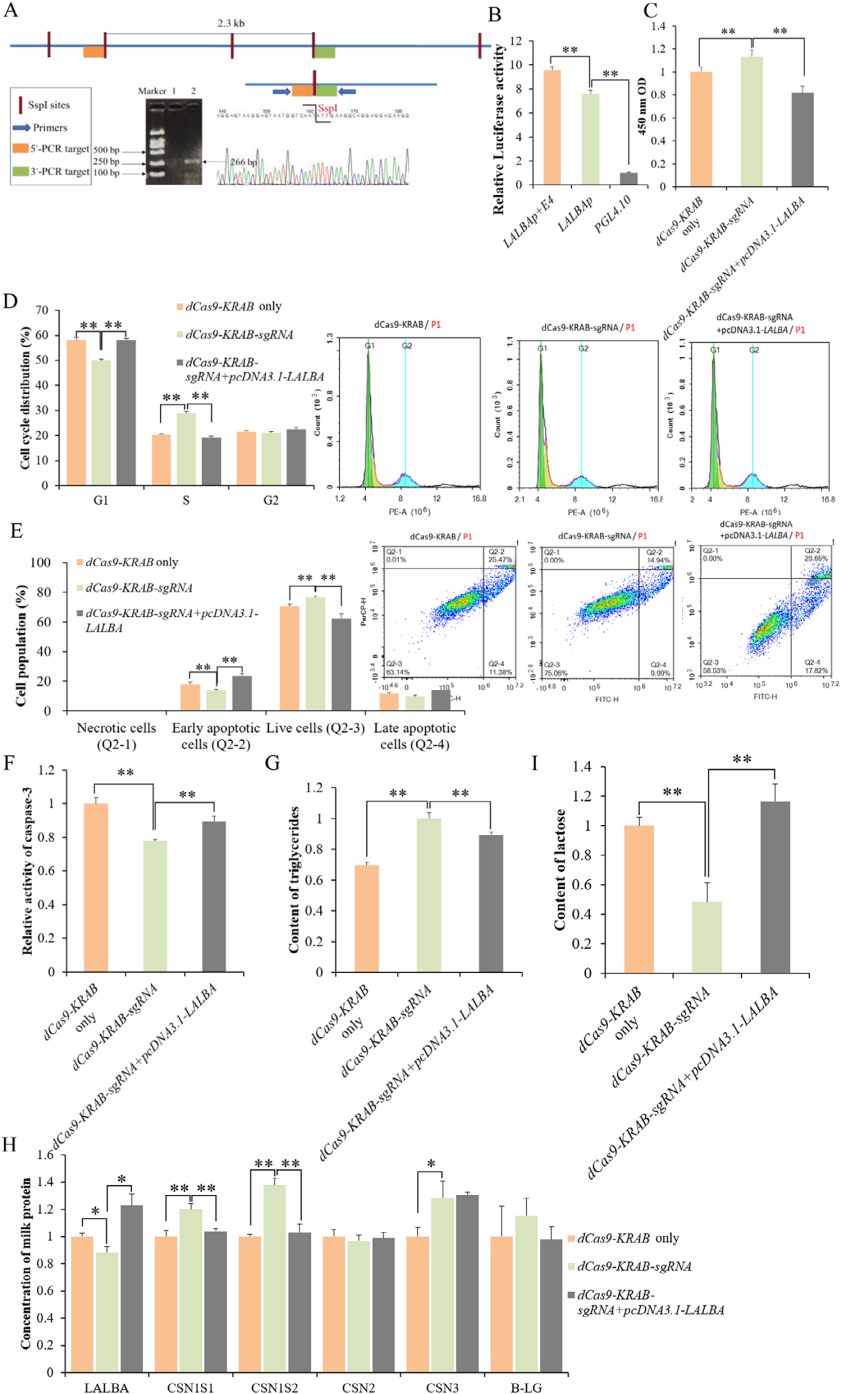
Effects of E4 on *LALBA* promoter and ovine mammary epithelial cells (OMECs). (A) Chromatin conformation analysis illustrated a loop linking E4 and the *LALBA* promoter. 1 represents un‐crosslinked DNA and 2 represents experimental group. (B) Effect of enhancer on activity of *LALBA* promoter. Effect of E4 on proliferation (C), cycle (D), apoptosis (E), caspase3 activity (F), triglycerides (G), milk protein (H) and lactose (I) of OMECs. Statistical significance was determined by one‐way ANOVA plus Tukey's post hoc tests in (B–I). All statistical data are represented as mean ± SD (*n* = 3; *p < 0.05, **p < 0.01).

Then the impact of E4 on the characteristics of OMECs was studied. CCK‐8 proliferation assays demonstrated that infected with dCas9‐KRAB‐sgRNA increased the proliferation of OMECs when compared to dCas9‐KRAB only (Figure 7C; *p* < 0.01). E4 induced cell arrested in the G0/G1 phase to delay the progression of the cell cycle (Figure 7D; *p* < 0.01). The Annexin‐V/PI assay was performed to examine the effect of E4 on apoptosis of OMECs. The flow cytometric analysis verified that apoptotic cell proportion was significantly decreased after infected cells (Figure 7E; *p* < 0.05). Consistent with flow cytometry data, E4 enhanced caspase 3 activity level in OMECs (Figure 7F; *p* < 0.01). These suggest that E4 inhibited the proliferation and cell cycle progression of OMECs, but it promoted its apoptosis. The triglycerides content of cell lines infected with targeted activity enhancer increased significantly (Figure 7G; *p* < 0.05). E4 significantly decreased the content of CSN1S1, CSN1S2, and CSN3 (*p* < 0.05), but it had no effect on CSN2 and B‐LG (Figure 7H). E4 increased the content of lactose in OMECs (Figure 7I; *p* < 0.01). The effect of inhibition of E4 activity on OMECs was alleviated after overexpressing *LALBA*.

### 2.8 E4 Recruits ELF5 to the *LALBA* Promoter for Enhancing *LALBA* Transcription to Decrease the Proliferation and Synthesis of Milk Fat of OMECs

To elucidate the upstream mechanism by which the enhancer regulated *LALBA* expression, we performed motif analysis within the *LALBA* super‐enhancer using the JASPAR database, and proved the *LALBA* super‐enhancer contained ELF5, STAT5, and GR‐binding motifs, suggesting potential involvement of these mammary‐enriched regulatory factors (File S10). ELF5 was selected for subsequent investigation based on its high motif score and expression level. We combined the ELF5 ChIP‐seq and ATAC‐seq data with histone ChIP‐seq data. ELF5 bound to ACRs of E1 and E4, it was further identified ELF5 bound differential ACRs of E4 and *LALBA* promoter (Figure 8A), and we verified that ELF5 could bind to these regions using ChIP‐qPCR (Figure 8B,C). ELF5 binds more strongly to E4 and promoter of *LALBA* in low lactation performance ewes, and this was consistent with the higher chromatin openness of E4 and *LALBA* promoter in low lactation performance ewes (Figure 8B,C), suggesting the potential formation of an enhancer‐TFs‐promoter complex. The dual‐luciferase reporter assay further confirmed that the *LALBA* promoter region bound by ELF5 is the core region (Figure 8D).

FIGURE 8E4 recruits ELF5 to the *LALBA* promoter, thereby enhancing *LALBA* expression and regulating cellular phenotypes. (A) Integrative Genomics Viewer (IGV) plot exhibited ELF5 bound to E4 and *LALBA* promoter in high (H) and low (L) lactation performance ewes. The *LALBA* promoter is defined as the region within 2 kb of the reference transcript start site (TSS). ChIP‐qPCR verified ELF5 bound to E4 (B) and *LALBA* promoter (C). (D) Luciferase assay showed that ELF5 was bound to the core region of the *LALBA* promoter. (E) Luciferase assays demonstrated that ELF5‐mediated loop between E4 and the *LALBA* promoter. The effect of *ELF5* on the expression of *LALBA* at mRNA (F) and protein level (G). Original uncropped blots can be found in Supplementary figure: “Original blots for (G)”. Effect of *LALBA* on proliferation (H), cycle (I), apoptosis (J), caspase3 activity (K), triglycerides (L), milk protein (M) and lactose (N) of cells. For statistical analysis, one‐way ANOVA plus Tukey's post hoc tests were performed in (D–E); two‐tailed unpaired Student's *t*‐tests were performed in (B–C) and (F–N). All statistical data are represented as mean ± SD (*n* = 3; * *p* < 0.05, ** *p* < 0.01).

**Figure:**
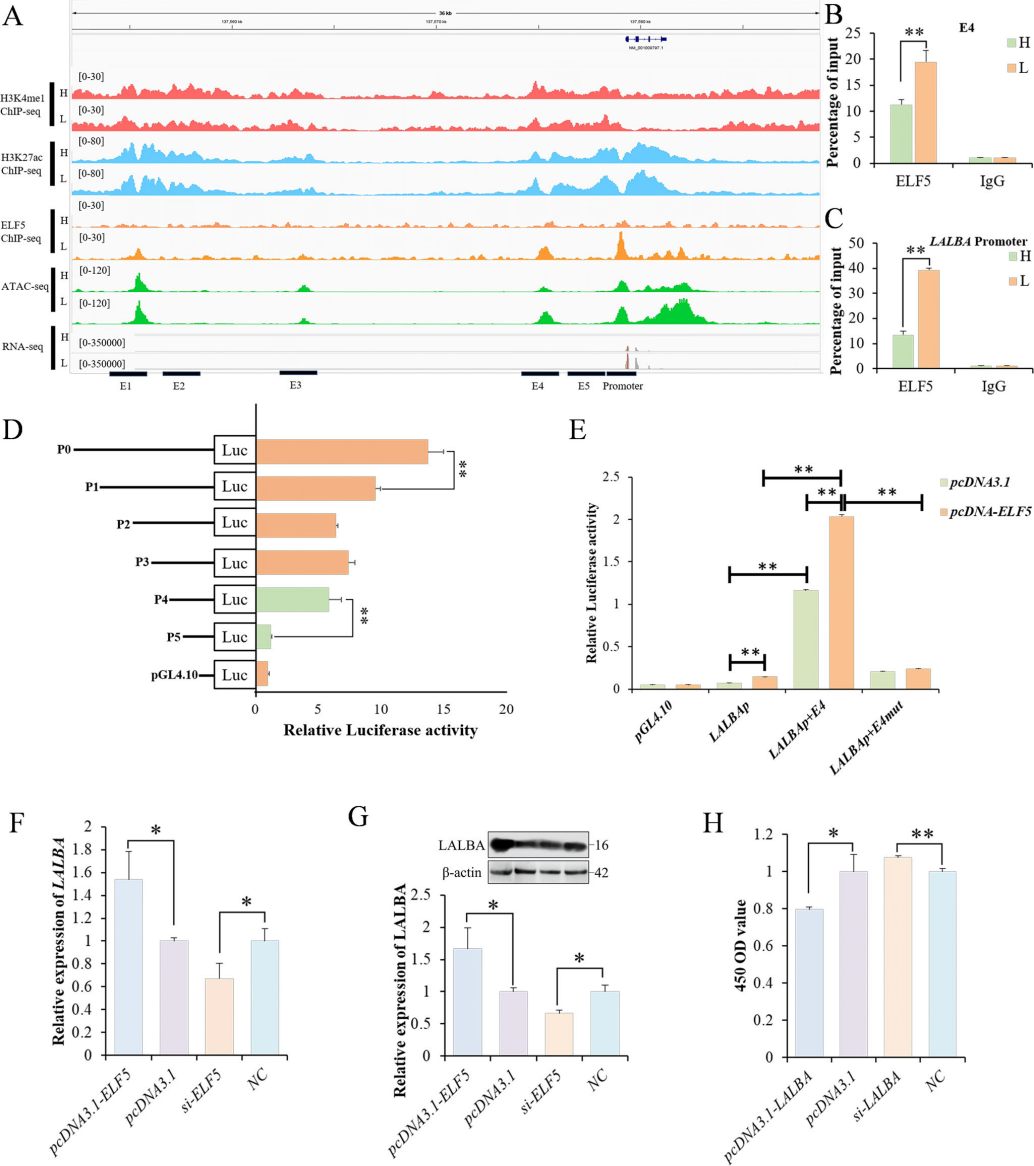

**Figure:**
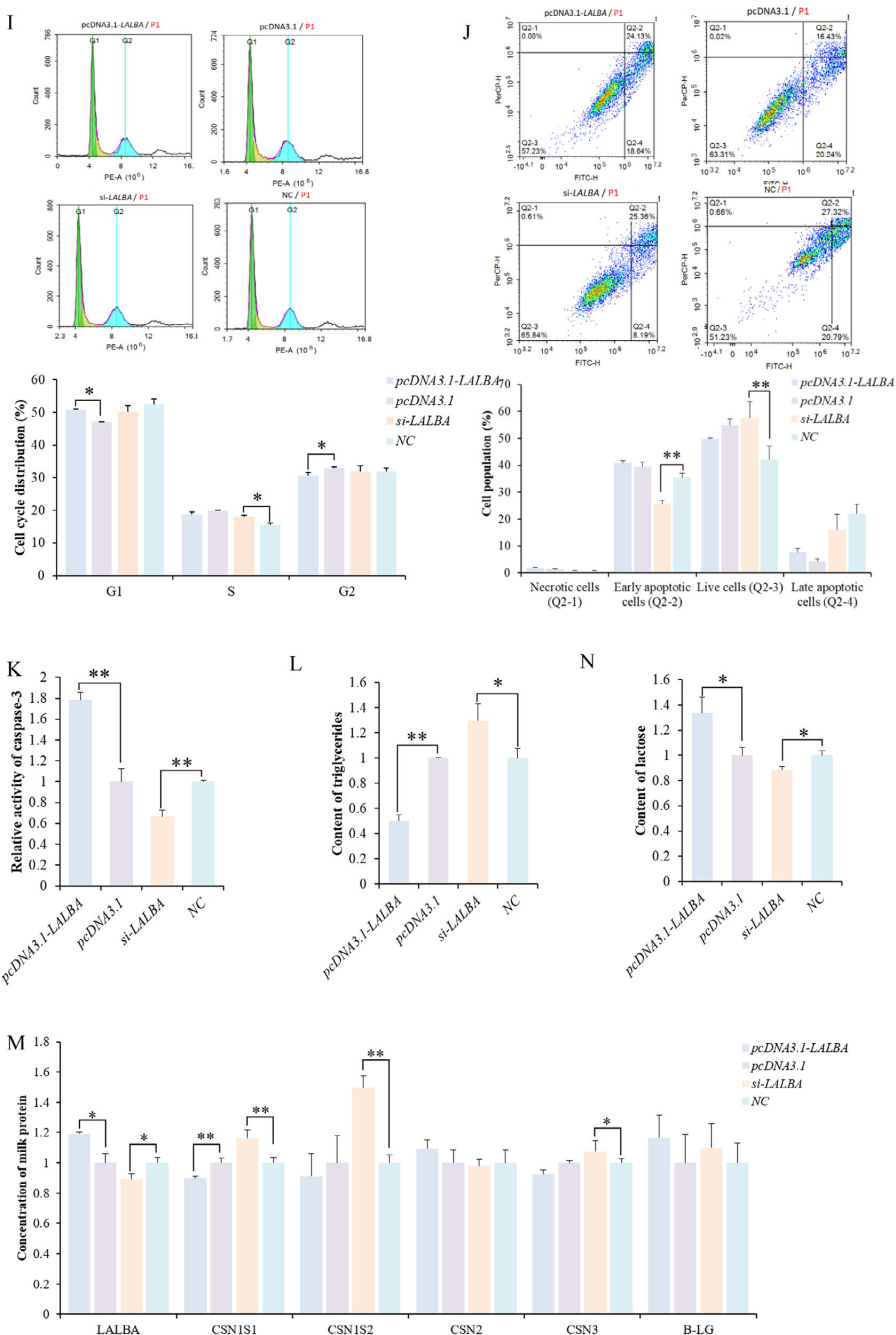

To investigate the role of ELF5 at the binding sites of E4 and the *LALBA* promoter, we conducted site‐directed mutagenesis on the cis‐regulatory sequences that contain GGAA‐box motifs. Mutation of the putative ELF5‐binding motifs in region E4 completely abolished enhancer activity and the response to ELF5 (Figure 8E and Figure S4A). Mutation of ELF5‐binding motifs in *LALBA* promoter also inhibited activity and the response to ELF5 (Figure S4B,C). The E4 region was responsive to ELF5 transactivation, which further increased luciferase activity compared with *LALBA* promoter alone (Figure 8E). These findings indicate that E4 can elevate *LALBA* promoter activity, which is further enhanced by ELF5. We further studied the regulatory effect of *ELF5* on *LALBA* through overexpressing and interfering the expression of *ELF5* in OMECs (Figure S4D). The RT‐qPCR and WB analysis indicated that overexpressed of *ELF5* significantly increased *LALBA* in expression (*p* < 0.05), while inhibitor of *ELF5* resulted in a decrease of *LALBA* expression in OMECs (Figure 8F; *p* < 0.05). These findings demonstrated that E4 and the *LALBA* promoter were physically and functionally connected through a ELF5‐mediated chromatin loop to enhance *LALBA* transcription.

Subsequently, the effect of *LALBA* on the characteristics of OMECs was studied. When pcDNA3.1‐*LALBA* and si‐*LALBA* were transfected into OMECs, RT‐qPCR and WB detection results showed that the mRNA and protein expression level of *LALBA* were significantly increased and decreased compared to its NC group, respectively (Figure S4E; *p* < 0.01). The CCK8 result showed that pcDNA3.1‐*LALBA* significantly decreased the proliferation of OMECs (*p* < 0.05), si‐*LALBA* showed the opposite results (Figure 8H; *p* < 0.05). PI flow cytometry results indicated that *LALBA* significantly increased the sub‐G1 cell proportion and decreased in both S and G2/M phase population in OMECs (Figure 8I). The flow cytometric analysis verified that apoptotic cell proportion was significantly decreased after knock down expression of *LALBA* (Figure 8J; *p* < 0.05). Consistent with flow cytometry data, *LALBA* enhanced caspase 3 activity level in OMECs (Figure 8K; *p* < 0.01). The content of triglycerides was significantly decreased in OMECs transfected with pcDNA3.1‐*LALBA* (*p* < 0.05), while si‐*LALBA* increased the content of triglycerides when compared with its NC group (Figure 8L; *p* < 0.05). To investigate the potential effect of *LALBA* to milk protein yield, the content of CSN1S1, CSN1S2, CSN2, CSN3, and B‐LG were measured in OMECs, *LALBA* exhibited identical biological functions of E4 (Figure 8M). *LALBA* increased the content of lactose in cells (Figure 8N; *p* < 0.05).

## 3 Discussion

Understanding the mechanisms regulating lactation performance in sheep may help solve the low survival and slow growth rates of multi‐born lambs caused by insufficient lactation of ewes, while also providing a theoretical basis for improving sheep breeding efficiency. The mammary gland acinus is the basic functional unit of milk production and consists of a single layer of OMECs. Therefore, the size and quantity of acinus determine the milk yield and milk composition of livestock [32, 33, 34, 35]. In this study, the area, diameter and circumference of acinus cavity were greater in high lactation ewes than in low lactation ewes. However, the regulatory mechanisms underlying the differences in ovine lactation performance remain to be elucidated.

Numerous genomic studies have shown that approximately 80% of non‐coding sequences are involved in regulating gene expression [36]. Epigenetics is based on the change of gene expression levels caused by non‐gene sequence changes, and chromatin accessibility is a major mode of epigenetic regulation [37, 38, 39]. We constructed the chromatin accessibility map of the ovine mammary gland for the first time to understand the genetic mechanism that caused the difference of lactation performance in sheep. Genome annotation revealed that ACRs were predominantly located in promoter, intron and intergenic region throughout the genome. This distribution was consistent with previous findings in the mammary gland of Holstein cows [40], mouse liver [41] and ovine skeletal muscle [42]. Genes associated with differential ACRs were enriched in pathways related to the negative regulation of T cell proliferation, insulin resistance, aldosterone synthesis and secretion signaling pathway. T cell proliferation has been implicated in mammary gland development [43]. The occurrence of insulin resistance led to a decrease of milk protein synthesis in mammary gland [44], while aldosterone can inhibit the conduction of insulin signal pathway and increase the occurrence of insulin resistance [45]. Therefore, chromatin remodeling in mammary gland led to differences in milk yield and milk composition between high lactation performance ewes and low lactation performance ewes.

The accessible genome comprises only about 2–3% of total DNA sequences, yet it captured more than 90% of TF‐binding regions. Consequently, chromatin accessibility provides an integrated reflection of TF occupancy and the regulatory potential of genomic loci [46, 47, 48]. The TFs that drove chromatin alterations in ovine mammary gland were identified, and the most TFs were found to belong to ETS family. ELF5 is a key regulator of mammary gland acinar differentiation and lactation function, as knocking out of ELF5 in mouse mammary gland led to acinar development defect and lactation failure [49]. It has been reported that ELF5 regulated mammary gland development through its target gene in mice. Rogers et al. [50] ascertained ELF5 binding to the Mucin 4 (*Muc4*) promoter in mouse mammary gland. In this study, ELF5 had the highest expression in ETS family, indicating that it may have vital biological function in the regulation of mammary gland development; we then subsequently focused on downstream experimental validation of ELF5.

ELF5 exhibited distinct preferences in gene binding between high‐ and low lactation ewes. The genes binding to ELF5 in high lactation performance ewes were significantly enriched in Rap1 signaling pathway, mTOR signaling pathway and Wnt signaling pathway. Rap1 and mTOR signaling pathways facilitated the synthesis of milk protein in the mammary gland [51, 52], while Wnt signaling pathway promoted mammary placode development and was essential for initiation of mammary gland morphogenesis [53]. In low lactation performance ewes, the target genes of ELF5 were related to the degradation of milk protein and milk fat. The binding sites of a given TF can vary substantially across cell types and physiological conditions [54]. Thus, differences in the intracellular environment of mammary gland between the two groups may contribute to the distinct ELF5‐binding profiles. In our results, ELF5 predominantly bound to intergenic and intron region of the genome, consistent with findings in mouse mammary gland tissues [55].

The differential ACRs and ELF5‐binding sites were enriched in intronic and intergenic regions, which frequently contain enhancers [56]. Therefore, we performed super‐enhancer analyses to further characterize the potential regulatory landscape associated with lactation performance. In this study, the region of *LALBA* super‐enhancer designated as E4 exerts a core function in driving the transcription activity of *LALBA*. E4 and *LALBA* promoter can be spatially closed by forming chromatin loops and three‐dimensional structures, so that transcription regulatory factors can play a more efficient role in functional sites. Previous studies generally confirmed that the regulation of enhancers showed a stronger transcription promotion effect than that of promoters alone [57, 58], and this difference can be attributed to the functions of enhancers in maintaining transcription activity, recruiting transcription complexes and amplifying the output signals of promoters [59, 60]. The enhancer promoted target gene expression through TF‐mediated enhancer‐promoter interactions [61, 62]. On this basis, we combined the binding site of ELF5 with the enhancer identified in mammary gland, and found that ELF5 binds to E4 and core promoter regions of *LALBA*. Moreover, ELF5 exhibited stronger binding to the E4 and the *LALBA* promoter in low lactation performance ewes. It was notably that both the E4 and promoter of *LALBA* displayed greater chromatin accessibility in low lactation performance ewes. Our results also confirmed again the proposition that alterations in the gene expression profile were accompanied by the redistribution of chromatin accessibility [63, 64, 65]. While dual‐luciferase reporter assays in this study have verified the activity of *LALBA* promoters in vitro, future studies integrating H3K4me3 ChIP‐seq would provide additional in vivo‐level evidence for characterizing promoter activity differences between the high and low lactation groups. The co‐location results at the level of space and cis‐element suggests that ELF5 participated in the synergistic regulation process of E4‐*LALBA* promoter, suggesting the potential formation of an enhancer‐TFs‐promoter complex. The double luciferase report assay further showed that ELF5 can significantly improve the level of enhancer‐driven promoter activity. These findings support that ELF5, as a key transcription regulator, mediated the functional coupling between E4 and *LALBA* promoter.

Cell‐based experiments showed that *LALBA* and E4 inhibited the proliferation and promoted the apoptosis of OMECs. Previous studies have shown that *LALBA* inhibited the proliferation and induced apoptosis of bovine mammary epithelial cells [66]. Sharp et al. [67] proved that *LALBA* induces apoptosis in mouse and human mammary epithelial cell lines, as well as in fur seal mammary epithelial cells. They proposed that apoptosis does not occur in fur seal mammary glands due to lack of *LALBA* in fur seal milk, allowing evasion of involution during a foraging trip, and identified *LALBA* as a milk factor that feeds back on the mammary gland to regulate involution. E4 and *LALBA* significantly decreased the content of triglycerides, CSN1S1, CSN1S2 and CSN3, and they significantly increased the content of lactose. The enhancer E4 may preferentially interact with and regulate the *LALBA* promoter through locus‐specific chromatin interactions, whereas *CSN2* expression no changed for it may be primarily controlled by its own enhancers and promoter elements. *LALBA*‐deficient mice produced a highly viscous milk that was rich in fat and protein but devoid of LALBA and lactose. The negative correlation between LALBA concentrations and content of protein and fat in milk was also observed in mice [68]. Although OMECs serve as a valid cellular model for mechanistic validation and for assessing effects on cellular characteristics, they cannot fully recapitulate the in vivo effects on lactation traits. Therefore, the in vivo validation will be required to determine the lactation performance in the future. In addition, the omics‐based predictions as exploratory candidates also requiring independent experimental validation. This study was conducted in a small sequencing cohort (*n* = 3 per group), which represents an important limitation. Accordingly, the molecular differences reported in this study are exploratory in nature, and replication in larger cohorts is required to confirm and extend these findings.

In summary, our work reveals the regulatory genome of sheep across mammary gland tissue. For the first time, we report the lactation performance differences derived from chromatin and uncover a potential regulatory mechanism focused around the role of ELF5 in the mammary gland. ELF5 bound to regulatory elements of genome and therefore ultimately influenced downstream genes transcriptional programs involved in lactation phenotype. The effect of the ELF5‐E4‐*LALBA* axis in regulating *LALBA* expression was particularly expounded. At the level of three‐dimensional genome, E4 is close to the *LALBA* promoter in spatial conformation, forming a functional chromatin loop, recruiting ELF5 and realizing the regulation of the spatial and temporal expression of *LALBA*. The lower chromatin openness in the E4 and promoter region of *LALBA* in the mammary gland of high lactation performance ewes resulted in lower occupancy of ELF5, these ultimately leading to lower expression level of *LALBA* in the mammary gland (Figure 9). These results will provide a new idea for molecular improvement of lactation performance of sheep.

**FIGURE 9:**
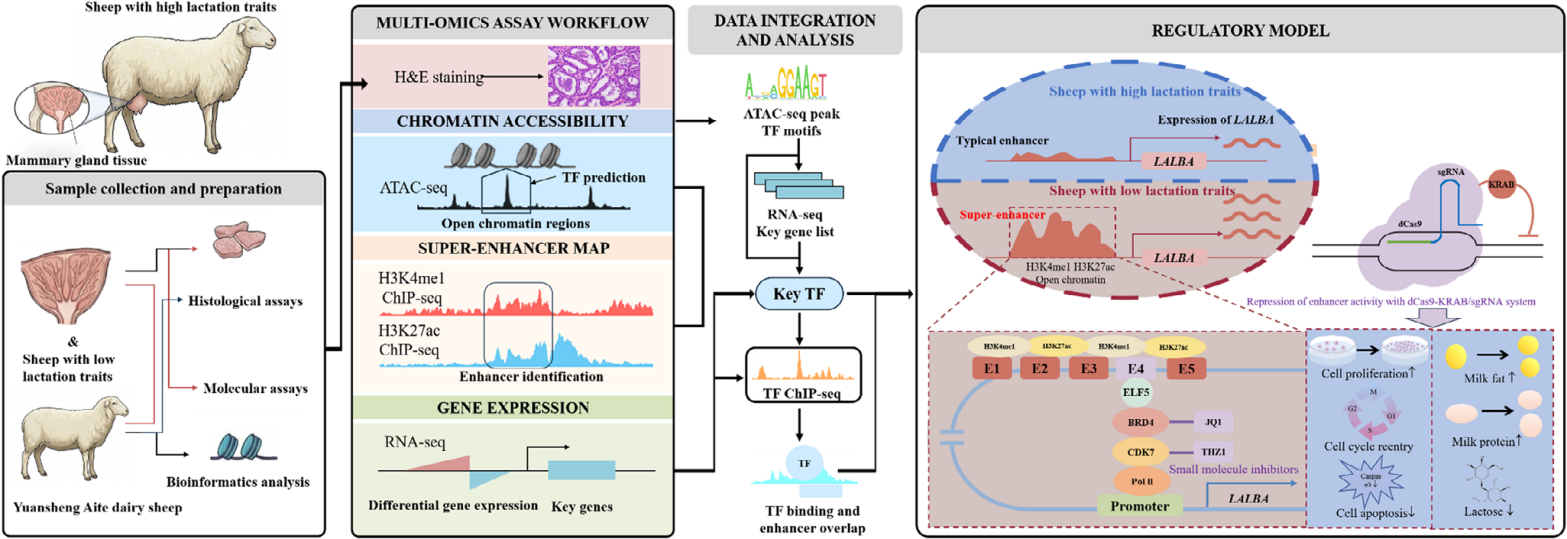
The mechanism of the regulatory axis of ELF5‐E4‐*LALBA* in the differences of lactation traits in sheep through multi‐omics integration analysis and functional verification.

## 4 Materials and Methods

### 4.1 Experimental Animals and Sample Collection

The experimental procedures involving animals in this study were approved by the Animal Experiment Ethics Committee of Gansu Agricultural University, Lanzhou, China (Approval number GSAU‐ETH‐AST‐2023‐029).

A total of 3473 Yuansheng Aite dairy sheep from the same farm of Gansu Yuansheng Animal Husbandry Technology Co., Ltd (Jinchang, China) were investigated as the basic group. To minimize the potential effects of parity on lactation performance, we restricted the analysis to 1362 primiparous ewes. From this population, 54 candidate Yuansheng Aite dairy sheep were selected that had the identical parity and litter size, similar in age and body weight at the mid‐lactation stage. All candidate ewes were in the same shed and maintained under the same management and diet conditions with ad libitum access to feed. Daily milk yield and days in milk were automatically recorded using a DeLaval milking system (DeLaval, Stockholm, Sweden). Milk fat and milk protein were measured using a milk composition analyzer (Youchuang, Hangzhou, China). Finally, the six primiparous, twin lambing, 1.5‐year‐old Yuansheng Aite dairy sheep that had extreme milk yield phenotypes were selected, as the ewes with higher milk yield also exhibited higher milk fat and protein percentages than those with lower milk yield, the ewes were further defined as high and low lactation performance ewes (Figure S5). The six ewes included three high lactation performance ewes (average milk yield: 1.72 ± 0.06 kg/d; average milk fat: 5.89 ± 0.39%; average milk protein: 5.72 ± 0.22% within 100–108 days postpartum) and three low lactation performance ewes (average milk yield: 1.10 ± 0.04 kg/d; average milk fat: 4.47 ± 0.18%; average milk protein: 5.14 ± 0.18% within 99–106 days postpartum) during mid‐lactation. Morphological indices describing the mammary glands of the six ewes were measured according to the method described by Margatho et al. [69]. Following the final milk collection, the ewes were slaughtered and their mammary gland tissues were collected for Hematoxylin‐Eosin (H&E) staining, ATAC‐seq, RNA‐seq, ChIP‐seq and culture of OMECs. The OMECs were cultured and identified using the method described previously by Liu et al. [70]. The detailed milk production performance of the ewes and morphological descriptions of their mammary gland are presented in Tables 1 and 2, respectively.

**TABLE 1:** Milk production performance of Yuansheng Aite dairy sheep with high and low lactation performance.

Group	Name	Age (month)	Lactation period (d)	Slaughter weight (kg)	Sig.	Average milk yield (kg/d)	Sig.	Average milk fat	Sig.	Average milk protein	Sig.
Low lactation performance ewes	L04	18.42	99	80.32	*p =* 0.570	1.16	*p =* 0.000	4.22	*p =* 0.009	5.40	*p =* 0.046
	L75	18.45	102	89.2		1.06		4.63		5.00	
	L00	18.42	106	75.99		1.07		4.55		5.03	
High lactation performance ewes	H76	18.45	100	72.98		1.74		5.86		5.57	
	H65	19.40	108	87.18		1.64		6.37		5.56	
	H86	18.45	107	74.28		1.79		5.43		6.03	

**TABLE 2:** The external morphology of mammary gland of Yuansheng Aite dairy sheep.

		Marmmary gland morphology									Nipple spacing (cm)	Sig.
Group	Name	Width (cm)	Height (cm)	Depth (cm)	Sig.	Length of central suspensory ligament (cm)	Sig.	Length of base plane (cm)	Circumference (cm)	Sig.		
Low lactation performance ewes	L04	14.5	17.2	7.0	*p =* 0.047	19.3	*p =* 0.040	9.5	41.3	*p =* 0.039	16.5	*p =* 0.482
	L75	15.2	14.8	9.5		19.5		10.0	44.4		16.5	
	L00	13.0	16.5	8.0		24.7		10.0	41.7		15.0	
High lactation performance ewes	H76	17.9	17.9	4.0		28.3		11.5	62.2		16.8	
	H65	14.5	18.4	6.2		25.2		10.5	49.5		15.2	
	H86	17.2	17.2	1.8		28.6		11.0	52.0		18.5	

### 4.2 H&E Staining

Mammary gland tissues from six ewes were used for histological analysis (three ewes per group). Each tissue samples were fixed with 4% paraformaldehyde for 48 h, followed by dehydration using graded ethanol solutions and embedding in paraffin. Three 5‐µm‐thick paraffin sections were prepared from each tissue sample, mounted on a glass slide for H&E staining. The slides were observed using Case Viewer (V.2.4). In this approach, nine visual fields were randomly selected, and the cross‐sectional area, diameter and circumference of each acinar cavity was measured with Motic Images Advanced (V.3.2).

### 4.3 ATAC‐seq and RNA‐seq

For ATAC‐seq, ATAC lysis buffer was added to the mammary gland tissue samples, and they were ground in a homogenizer. Cell nuclei were collected after filtering with a cell sieve. Following blending with transpositional reaction solution with the nuclear precipitate, the mixture was subjected to incubation at 37°C for 30 min and then purified with 2× DNA clean beads (Vazyme, Nanjing, China). Transpositional fragments were amplified by PCR for 15 cycles using the TruePrep Index Kit V2 for Illumina (Vazyme, Nanjing, China) with indexed adapter primers. Transposition and high‐throughput DNA sequencing libraries were then constructed using the TruePrep DNA Library Prep Kit V2 for Illumina (Vazyme, Nanjing, China). The library products were enriched, quantified and sequenced on a DNBSEQ‐T7 sequencer (MGI Tech Co., Ltd, Shenzhen, China) using the Paired‐End 150 bp (PE150) configuration.

For RNA‐seq, total RNA from the mammary gland tissues were extracted using Trizol Reagent Kit (Invitrogen, CA, USA). The concentration and purity of RNA were assessed using a NanoDrop spectrophotometer (Thermo Scientific, MA, USA), and the integrity of RNA was evaluated using an Agilent 2100 Bioanalyzer (Agilent, CA, USA). Only RNA extracts with an integrity number greater than 7 were used for library preparation. A total of 2 µg of the high‐quality RNA was used for RNA sequencing library preparation using the KC‐Digital Stranded mRNA Library Prep Kit for Illumina (Wuhan Seq health Co., Ltd, Wuhan, China). To eliminate duplication bias introduced by PCR amplification and sequencing steps, unique molecular identifiers (UID) that carrying 8–10 bp random sequences were ligated to cDNA molecules using the KC‐Digital RNA Library Prep Kit (Wuhan Seq health Co., Ltd, Wuhan, China). The library products were enriched, quantified, and finally sequenced on DNBSEQ‐T7 sequencer (MGI Tech Co., Ltd, Shenzhen, China).

### 4.4 Analysis of ACRs and Transcriptional Profiling in Ovine Mammary Gland

Raw sequencing data was filtered by Trimmomatic (V.0.36), and clean reads were further treated with FastUniq (V.1.1) to eliminate duplication. Clean data was mapped against the ovine reference genome ARS‐UI_Ramb_V.3.0 using Bowtie2 (V.2.2.6). ATAC‐seq peaks were detected using MACS2 (V.2.2.7.1) and then analyzed by IDR (V.2.0.3) to generate peaks with high reliability (screening parameters: IDR = 0.05, peak score ≥540). To avoid bias arising from separate peak calling, we generated a non‐redundant consensus ACRs set by merging ACRs identified across both groups. Csaw (V.1.40.0) based on edgeR was used to analyze the differential peaks in mammary gland tissues between high lactation performance ewes and low lactation performance ewes. ACRs meeting both criteria of Fold Change >2 and unadjusted *p* < 0.05 were considered differentially ACRs [71]. The differential peaks between the two groups were visualized using Integrative Genomics Viewer (IGV) tool (V.2.19.5). Bedtools (V.2.25.0) was employed to analyze the annotation and distribution of peaks, and the footprint analysis of TFs in ACRs was also performed. Homer (V.5.1) was used for motifs analysis and ACRs annotation. Homer assigns each ACR to the gene associated with its nearest annotated TSS and reports the corresponding distance; it also classifies the ACR according to its genomic context, including promoter‐TSS, exon, intron, and intergenic regions. Promoter is defined as the region within 2 kb of the reference TSS [72]. The unique nearest genes associated with ACRs showing increased and decreased accessibility were subsequently subjected to functional enrichment analysis. GO enrichment analysis was performed to classify genes into three major functional categories, including biological process, cellular component, and molecular function. KEGG pathway analysis was conducted to identify significantly enriched biological pathways. Enriched GO terms and KEGG pathways with *p* < 0.05 were considered significant.

Raw sequencing data was filtered by Trimmomatic (V.0.36). Low‐quality reads were discarded and the reads contaminated with adaptor sequences were trimmed. The clean reads were mapped against the ovine reference genome ARS‐UI_Ramb_V 3.0 using Bowtie2 (V.2.2.6). The reads per kilobase per million reads (RPKM) was used to normalize gene expression. DEGs between the two groups were identified by DESeq2 package (V.1.36.0) in the R program. The low lactation performance group was specified as the reference group, and the contrast was defined as high versus low lactation performance. Genes with Fold Change >1.5 and unadjusted *p* < 0.05 were considered to be significant DEGs [73]. The functional annotation of DEGs was performed with GO and KEGG databases. A total of 12 DEGs were randomly selected for RT‐qPCR to verify the results of RNA‐seq, and *β‐actin* was used as an internal reference. The primer information for these DEGs is listed in Table S1. All primers used in this study were synthesized by Sangon Biotech (Shanghai, China).

Pearson correlation analysis was performed to evaluate the relationship between differences in gene expression and changes in chromatin accessibility using GraphPad Prism (V.8.0). The Pearson correlation coefficient (r) and corresponding *p*‐value were calculated.

### 4.5 ChIP‐seq and Data Analysis

Mammary gland tissues were ground and then treated in 1% buffered formaldehyde solution for 15 min to cross‐link the protein and DNA. Glycine to 0.125 M was added to terminate the reaction. The ground powder was treated with cell lysis buffer, and nuclei were collected by centrifuging at 2000× *g* for 5 min. These nuclei were treated with lysis buffer and sonicated to fragment the chromatin. The antibody including ELF5 (Thermo Scientific, MA, USA; Cat. No.720380), H3K4me1 (CST, MA, USA; Cat. No.5326) and H3K27ac (Abcam, Cambridge, UK; Cat. No. ab4729) were selected for ChIP‐seq. The high‐throughput DNA sequencing libraries were prepared using a VAHTS Universal DNA Library Prep Kit for Illumina V3 sequencing (Vazyme, Nanjing, China). The library products corresponding to 200–500 bp were enriched by magnetic beads (Beckman Coulter, CA, USA), quantified via Qubit fluorometry (Thermo Scientific, MA, USA), and then sequenced with a DNBSEQ‐T7 sequencer (MGI Tech Co., Ltd. China) using the Paired‐End 150 bp (PE150) configuration. Specific primers were designed for the candidate ELF5‐binding regions and histone modification enrichment regions identified by ChIP‐seq. ChIP‐qPCR was performed to validate the occupancy of ELF5 and histone marks at the target genomic regions using SimpleChIP Universal qPCR Master Mix (CST, MA, USA; Cat. No.88989). The efficiency of the immunoprecipitation (IP) was calculated using the percentage of input method (%Input = 100×2^(Ct Input−Ct IP)^). Primers are listed in Table S2.

Raw sequencing data was filtered using Trimmomatic (V.0.36), and clean reads were then mapped to the ovine reference genome assembly ARS‐UI_Ramb_V.3.0 using STAR software (V.2.5.3). RSeQC (V.2.6) was used for reads distribution analysis. Peak calling was performed using MACS2 software (V.2.2.7.1). Bedtools (V.2.25.0) was used to undertake peak annotation and distribution analysis. Peak maps of reads across the genome were described using IGV tool (V.2.19.5). The differentially binding peaks between the two groups were identified using the Csaw (V.1.40.0) based on the edgeR statistical framework, and the differential peaks were screened with Fold Change >2 and unadjusted *p* < 0.05 [71].

### 4.6 Identification of Super‐Enhancers and Repression of Enhancer Activity With dCas9‐KRAB/sgRNA System

The overlapping H3K4me1 and H3K27ac peaks were potential enhancers. If they were separated by an interval of less than 12.5 kb, they were connected and clustered into super‐enhancers. Super‐enhancer and enhancer calling was undertaken using Rank Ordering of Super‐enhancers (ROSE) software (V.1.0) based on H3K27ac enrichment using the default parameters. A tangent slope of 1 was used as the threshold to determine the inflection point of the rank‐ordering curve, and regions above the inflection point of the rank‐ordering curve were defined as super‐enhancers. For the identified enhancers and super‐enhancers, the nearest gene was selected as a potential target for the enhancer elements. ACRs identified by ATAC‐seq were integrated with H3K27ac and H3K4me1 ChIP‐seq signals to define candidate active enhancers.

The single guide RNA (sgRNA) sequences were designed using the CRISPR design tool (http://tools.genome‐engineering.org). The target sequences of the ovine *LALBA* enhancer sgRNAs were as follow: sgRNA1 5′‐ATGGAACTTGATCTTGCTGA‐3′, sgRNA2 5′‐TGAGTAACTGATAGAGACCT‐3′, sgRNA3 5′‐TTGAGCCAGATCTTGGCCTG‐3′. The oligo pairs containing the 20‐nt guide sequences were annealed and ligated into the dCas9‐KRAB‐T2a‐Puro plasmid. These plasmids were transfected into human embryonic kidney 293T (HEK293T) cells using a Calcium Phosphate Cell Transfection Kit (Beyotime, Shanghai, China; Cat. No. C0508), the culture medium containing lentiviral particles was collected after transfected for 48 h, and it was concentrated using the Universal Virus Concentration Kit (Beyotime, Shanghai, China; Cat. No. C2901L). The titer of lentiviral particles measured and adjusted to a titer of 1.25 × 10^8‐9^ TU/mL by the Rapid Lentivirus Titration Cassette (Biodragon, Suzhou, China; Cat. No. BF06202). OMECs were infected with the concentrated lentiviral particles, which were then selected with 2 µg/mL puromycin (Beyotime, Shanghai, China; Cat. No. ST551) to eliminate non‐transduced cells.

### 4.7 Chromosome Conformation Capture (3C) Assay

3C assay was performed using a commercial 3C assay kit (BersinBio, Guangzhou, China; Cat. No. Bes5006) according to the manufacturer's instructions. Briefly, approximately 1 × 10^7^ OMECs were crosslinked with formaldehyde at a final concentration of 1% at room temperature for 10 min using a vertical mixer. The crosslinking reaction was terminated by adding glycine (final concentration 125 mM) and incubating for 5 min at room temperature. OMECs were collected by centrifugation at 2000 rpm at 4°C. The crosslinked cells were lysed in lysis buffer supplemented with protease inhibitor and incubated on ice for 15 min. After mechanical disruption, precipitated particles were collected by centrifugation and washed with 1× restriction enzyme buffer. The nuclei were incubated in restriction enzyme buffer and treated with 200 U *Ssp*I restriction enzyme (Beyotime, Shanghai, China) at 37°C overnight. The digested nuclei were subjected to proximity ligation in a dilute reaction mixture containing T4 DNA ligase and incubated at 16°C for 2 h. The ligation products were then treated with 20 µL proteinase K and incubated at 65°C overnight. The DNA was extracted and amplified according to the instruction manual of 3C assay kit (BersinBio, Guangzhou, China), and the primers are listed in Table S1. The PCR products were analyzed by agarose gel electrophoresis to verify the expected amplicon size and subsequently confirmed by Sanger sequencing.

### 4.8 Overexpression Vector Construction and Treatment of OMECs

To construct the pcDNA3.1‐*ELF5* and pcDNA3.1‐*LALBA* vectors, the full‐length sequences of ovine *ELF5* and *LALBA* CDS were amplified using cDNA obtained from mammary gland tissue collected originally and then sequenced by Synbio Co., Ltd (Suzhou, China). The full‐length sequence of ovine *ELF5* and *LALBA* CDS were connected to pcDNA3.1 vector (Synbio, Suzhou, China) between the *Hind*III and *EcoR*I sites, respectively. Plasmid DNA were extracted from pcDNA3.1‐*ELF5* and pcDNA3.1‐*LALBA* vectors using the EndoFree Midi Plasmid Kit (Tiangen, Beijing, China). The si‐*ELF5* and si‐*LALBA* were synthesized in GenePharma Co., Ltd (Shanghai, China). The sequences of the siRNAs used in this study are listed in Table S3.

When the density of OMECs cultured in 12‐well plates using whole culture medium (DMEM/F12 medium, 10% fetal bovine serum, 5 µg/mL prolactin and 5 µg/mL insulin) was 70–80%, pcDNA3.1‐*ELF5*, pcDNA3.1‐*LALBA*, si‐*ELF5* and si‐*LALBA* were individually transfected into OMECs using INVI DNA&RNA Transfection Reagent (Invigentech, CA, USA). After transfection for 48 h, total RNA was isolated using Trizol Reagent (Invitrogen, CA, USA), and then reverse transcribed to generate cDNA using Super ScriptTM II reverse transcriptase (Invitrogen, CA, USA). The expression levels of *ELF5* and *LALBA* were calculated using RT‐qPCR and *β‐actin* was used as an internal reference. For experiments using inhibitors, cells well treated with JQ‐1 (MCE, NJ, USA) and THZ1 (MCE, NJ, USA) for 6 h, and their effect on expression of *LALBA* was detected. The primers are listed in Table S1.

### 4.9 Luciferase Reporter Vector Construction and Dual Luciferase Reporter Assay

The sequence of *LALBA* super‐enhancer elements was amplified by PCR from genomic DNA extracted from Yuansheng Aite dairy sheep and ligated into the pGL3‐promoter vector (Synbio, Suzhou, China) between the *Kpn*I and *Sma*I sites to verify the activity of enhancers. A 2.3 kb DNA fragment containing the *LALBA* promoter, and five promoter deletion constructs designed to delete different upstream regions of the promoter were ligated into the pGL4.10 luciferase reporter plasmid (Synbio, Suzhou, China) between the *Kpn*I and *Xho*I sites, then the *LALBA* enhancer was cloned into *BamH*I and *Sal*I sites of pGL4.10 located approximately 2 kb upstream of the *LALBA* promoter insert. The primer information is listed in Table S4. *LALBA* enhancer and promoter sequences containing the mutation of the ELF5 binding sites were commercially synthesized (Synbio, Suzhou, China).

For transactivation assays, the *LALBA* enhancer and promoter reporter constructs were co‐transfected into HEK293T along with Renilla luciferase and either pcDNA3.1‐*ELF5* or pcDNA3.1 plasmid. Luciferase activity was detected using the Dual‐Luciferase Reporter Kit (Promega, WI, USA; Cat. No. E1910) and a Varioskan LUX Microplate Reader (Thermo Scientific, MA, USA).

### 4.10 Western Blotting

Total protein was extracted using RIPA buffer (Solarbio, Beijing, China). Protein concentrations were quantified with the BCA Protein Assay Kit (Solarbio, Beijing, China). The samples were loaded onto SDS‐PAGE gels (Epizyme, Shanghai, China) for electrophoresis and then transferred to polyvinylidene fluoride membranes (Biosharp, Hefei, China) for immunoblotting. Subsequently, the membranes were placed in protein‐free rapid blocking buffer (Epizyme, Shanghai, China) and incubated for 10 min, followed by overnight incubation at 4°C with the primary antibody. After washing the membrane with Tris‑Buffered Saline with Tween‑20, the membrane was incubated with secondary antibody at 37°C for 1 h. Following treatment with Enhanced Chemiluminescence (ECL) western blotting substrate (Biosharp, Hefei, China), and the chemiluminescence signal was detected with a chemiluminescence imaging system (Vilber, Paris, France). Band quantification was performed using ImageJ software V1.8.0 (NIH, Bethesda, MD, USA). The primary antibodies used in the study included ELF5 antibody (Thermo Scientific, MA, USA; Cat. No.720380), LALBA (Zenbio, Chengdu, China; Cat. No.250044) and β‐actin (Proteintech, Wuhan, China; Cat. No.66009‐1‐Ig), while the secondary antibody contained HRP‐conjugated Goat Anti‐Rabbit IgG (H + L) (Proteintech, Wuhan, China; Cat. No. SA00001‐2) and HRP‐conjugated Goat Anti‐Mouse IgG (H + L) (Proteintech, Wuhan, China; Cat. No. SA00001‐1).

### 4.11 Effect of E4 and *LALBA* on Proliferation and Cell Cycle Progression of OMECs

A total of 10 µL of CCK‐8 reagent (Vazyme, Nanjing, China; Cat. No. A311‐01) was supplemented to each well of 96‐well plates, followed by incubation at 37°C for 2 h. The absorbance was measured at 450 nm using a microplate reader (Thermo Scientific, MA, USA).

OMECs cultured in plates were harvested after digestion with pancreatin (Solarbio, Beijing, China). The cell suspensions were collected by centrifugation and then fixed in 70% ethanol at 4°C for 12 h. After 200× *g* centrifugation for 3–5 min, the ethanol solution was removed, and the cell precipitate retained in the centrifuge tube. Staining solution containing propidium iodide (PI) and RNase A was prepared according to instructions (Servicebio, Wuhan, China; Cat. No. G1700) and incubated at 37°C for 30 min. The stained cells were analyzed using Flow Cytometry (Agilent, Beijing, China), and the percentages of cells in the G0/G1, S, and G2/M phases were calculated using flow cytometry analysis software.

### 4.12 Effect of E4 and *LALBA* on Apoptosis of OMECs

According to the instructions of Annexin V‐FITC/PI Apoptosis Detection Kit (MeilunBio, Dalian, China; Cat. No. MAO220), 1× Annexin V binding buffer was added to the cell precipitation. A total of 5 µL of Annexin V‐FITC and 5 µL of PI were added to cells and incubated for 15 min. Subsequently, 1× Annexin V binding buffer solution was added. The apoptosis of cells was detected by Flow Cytometry (Agilent, Beijing, China), and the percentages of apoptotic cells were calculated.

To evaluate apoptosis‐related Caspase‐3 activity, 100 µL of cell lysate was added to cells, followed by lysis on ice for 30 min. During lysis, vortex oscillation was performed 3–4 times. Subsequently, centrifugation was conducted at 12 000 rpm for 15 min, and the supernatant was collected to measure Caspase 3 enzyme activity according to Caspase 3 activity detection kit (Multi Sciences, Hangzhou, China; Cat. No. APC03).

### 4.13 Effect of E4 and *LALBA* on Synthesis of Milk Fat, Protein, and Lactose of OMECs

The OMECs were washed using PBS and collected into a centrifuge tube. Cells were lysed by ultrasonication, and the lysate was centrifuged at 8000× *g* for 10 min to collect the supernatant for analysis. The concentration of triglyceride in the supernatant was detected using a Triglyceride Content Detection Kit (Solarbio, Beijing, China; Cat. No. BC0620). The absorbance was measured at 420 nm using a microplate reader (Thermo Scientific, MA, USA), and the triglycerides concentration was calculated based on the standard curve generated with the triglyceride standard.

According to the instructions of ovine LALBA ELISA Kit (Byabscience, Nanjing, China; Cat. No. BY‐ES779131), β‐casein (CSN2) ELISA Kit (Byabscience, Nanjing, China; Cat. No. BY‐ES779297), αs1‐casein (CSN1S1) ELISA Kit (Byabscience, Nanjing, China; Cat. No. BY‐ES779088), αs2‐casein (CSN1S2) ELISA Kit (Mlbio, Shanghai, China; Cat. No. YJ320332B), kappa‐casein (CSN3) ELISA Kit (Mlbio, Shanghai, China; Cat. No. YJ293021B) and β‐lactoglobulin (B‐LG) ELISA Kit (Byabscience, Nanjing, China; Cat. No. BY‐ES779132), the content of milk protein that mentioned above was detected. Lactose content was determined using the Lactose Content Determination Kit (Biosharp, Hefei, China; Cat. No. BL1797B) according to the manufacturer's instructions. The absorbance of the preparation was detected at 510 nm using a microplate reader (Thermo Scientific, MA, USA).

### 4.14 Statistical Analysis

All analyses were performed using SPSS (V.25.0). Data are presented as mean ± standard deviation (SD), as specified in the corresponding figure legends. Statistical analyses were performed using two‐tailed unpaired Student's *t*‐tests or one‐way ANOVA followed by Tukey's post hoc test for multiple comparisons. For all experiments, *p* < 0.05 was considered significant (*p < 0.05, **p < 0.01). Sample sizes (*n*) indicated in the Figure legends represent biological replicates. All experiments were independently repeated at least three times, and representative data were shown.

## Author Contributions

**Xinmiao Wu**: writing – original draft, investigation, validation, visualization. **Jiqing Wang**: writing – review & editing, funding acquisition, conceptualization, data curation, project administration, resources, supervision. **Zhiyun Hao**, **Mingna Li**, **Chunyan Ren** and **Xuelan Zhou**: software, collected the samples. **Huimin Zhen**, **Yuan Liu**, **Guoyong Zhao**, **Haixiong Hu**, **Mingjie Li**, and **Nan Shi**: investigation, sample collection. All authors reviewed and approved the final version.

## Funding

This work was financially supported by the National Natural Science Foundation of China (32472879), the Key Research and Development Program of Gansu Province (25YFNA029), the Seed Industry Research Project of Gansu Province (ZYGG‐2026‐3), the earmarked fund for Gansu Agriculture Research System (GSARS02‐05), the Projects of Gansu Animal Husbandry General Station (GSAU‐JSZX‐2024‐003), and the Discipline Team Project of Gansu Agricultural University.

## Conflicts of Interest

The authors declare no conflicts of interest.

## Supporting information




**Supporting File**: advs78138‐sup‐0001‐SuppMat.zip.

## Data Availability

The sequencing data used in this study had been deposited in the SRA database under project number PRJNA1234851 (ATAC‐seq), PRJNA1291917 (ELF5 ChIP‐seq), PRJNA1232630 (H3K4me1 ChIP‐seq, H3K27ac ChIP‐seq) and PRJNA1275754 (RNA‐seq).
