# Supplementary material for: ELF5‐Mediated Enhancer–Promoter Interaction Regulates LALBA Expression in Ovine Mammary Gland
Source: Adv Sci (Weinh). 2026 Oct 4:e78138. Online ahead of print. doi: 10.1002/advs.78138 (PMC13634586; doi:10.1002/advs.78138)
Supplement: Supplementary file 1 [file ADVS-9999-e78138-s001.zip › Supporting figures.docx]

**Supporting Figures**

A

B

**H**

**L**

C

Feature distribution

|  | promoter-tss | intergenic | other intron | other exon | 1st intron | tts | 1st exon |
| --- | --- | --- | --- | --- | --- | --- | --- |
| L | 9161 | 4933 | 4127 | 671 | 628 | 253 | 169 |
| H | 11146 | 8294 | 7931 | 1329 | 1191 | 448 | 334 |

D

**Figure S1.** Chromatin accessibility characteristics for mammary gland of Yuansheng Aite dairy sheep with high (H) and low (L) lactation performance. (A) The distribution (top) and heatmap (bottom) of reads relative to transcription start sites (TSS). (B) The distribution profiles of peaks relative to TSS. (C) The distribution of the genomic location of peaks. (D) Percentage of peaks located within upstream and downstream from the TSS of their nearest genes.

A

B

C

**Figure S2.** The screening process of super-enhancer associated genes. (A) Unique super-enhancer in mammary gland. (B) Effect of small molecular inhibitors on the expression of super-enhancer related gene *LALBA*. J100, treatment with 100 nM JQ1; T100, treatment with 100 nM THZ1. J0 and T0 represent untreated cells and therefore reflect the basal expression level of *LALBA* under the corresponding experimental conditions. (C) The expression of *LALBA* mRNA (Left) and protein (Right) under conditions of inhibited enhancer activity or simultaneous overexpression of *LALBA*. Original uncropped blots can be found in Supplementary figure: “Original blots for Figure S2C”. All statistical data are represented as mean ± SD (*n* = 3). Statistical significance was determined by two-tailed unpaired *t*-test in B and one-way ANOVA plus Tukey's post hoc tests in C. ** *P* < 0.01.

**Figure S3.** Effect of enhancer on activity of *LALBA* promoter. The statistical data are represented as mean ± SD (*n* = 3). Statistical significance was determined by one-way ANOVA plus Tukey's post hoc tests. ** *P* < 0.01.

A

B

C

D

E

**Figure S4.** ELF5 bound to the enhancer and promoter of *LALBA* and positively regulated the expression of *LALBA*. Effect of mutations in the ELF5 binding sites within the E4 (A) and promoter (B) on their activities. (C) Effect of ELF5 on *LALBA* promoter activity. Relative expression level of *ELF5* (D) and *LALBA* (E) when pcDNA3.1-*ELF5*, pcDNA3.1-*LALBA*, si-*ELF5* and si-*LALBA* were transfected into ovine mammary epithelial cells (OMECs), respectively. Original uncropped blots can be found in Supplementary figure: “Original blots for Figure S4D” and “Original blots for Figure S4E”. All statistical data are represented as mean ± SD (*n* = 3). Statistical significance was determined by two-tailed unpaired *t*-test. * *P* < 0.05, ** *P* < 0.01.

**Figure S5.** Flowchart of animal selection and definition of high and low lactation performance groups.
