# Supplementary material for: ELF5‐Mediated Enhancer–Promoter Interaction Regulates LALBA Expression in Ovine Mammary Gland
Source: Adv Sci (Weinh). 2026 Oct 4:e78138. Online ahead of print. doi: 10.1002/advs.78138 (PMC13634586; doi:10.1002/advs.78138)
Supplement: Supplementary file 1 [file ADVS-9999-e78138-s001.zip › ~WRL3004.tmp]

**Supporting Figures**

A

B

C

**H**

**L**

D

Feature distribution

|  | promoter-tss | intergenic | other intron | other exon | 1st intron | tts | 1st exon |
| --- | --- | --- | --- | --- | --- | --- | --- |
| L | 9161 | 4933 | 4127 | 671 | 628 | 253 | 169 |
| H | 11146 | 8294 | 7931 | 1329 | 1191 | 448 | 334 |

E

**Figure S1.** Chromatin accessibility characteristics for mammary gland of Yuansheng Aite dairy sheep with high (H) and low (L) lactation performance. (A) Distribution of DNA fragment length in libraries. (B) The distribution (top) and heatmap (bottom) of reads relative to transcription start sites (TSS). (C) The distribution profiles of peaks relative to TSS. (D) The distribution of the genomic location of peaks. (E) Percentage of peaks located within upstream and downstream from the TSS of their nearest genes.

A

B

C

β-actin

LALBA

**Figure S2.** The screening process of super-enhancer associated genes. (A) Unique super-enhancer in mammary gland. (B) Effect of small molecular inhibitors on the expression of super-enhancer related gene *LALBA*. J100, treatment with 100 nM JQ1; T100, treatment with 100 nM THZ1. J0 and T0 represent untreated cells and therefore reflect the basal expression level of *LALBA* under the corresponding experimental conditions. (C) The expression of *LALBA* mRNA (Left) and protein (Right) under conditions of inhibited enhancer activity or simultaneous overexpression of *LALBA*. ** *P* < 0.01.

**Figure S3.** Effect of enhancer on activity of *LALBA* promoter.

A

B

C

D

E

**Figure S4.** ELF5 bound to the enhancer and promoter of *LALBA* and positively regulated the expression of *LALBA*. Effect of mutations in the ELF5 binding sites within the E4 (A) and promoter (B) on their activities. (C) Effect of ELF5 on *LALBA* promoter activity. Relative expression level of *ELF5* (D) and *LALBA* (E) when pcDNA3.1-*ELF5*, pcDNA3.1-*LALBA*, si-*ELF5* and si-*LALBA* were transfected into ovine mammary epithelial cells (OMECs), respectively. * *P* < 0.05 and ** *P* < 0.01.

**Figure S5.** Flowchart of animal selection and definition of high and low lactation performance groups.
